# Application of Solution-Processed High-Entropy Metal Oxide Dielectric Layers with High Dielectric Constant and Wide Bandgap in Thin-Film Transistors

**DOI:** 10.3390/mi15121465

**Published:** 2024-11-30

**Authors:** Jun Liu, Xin Xiong, Han Li, Xiangchen Huang, Yajun Wang, Yifa Sheng, Zhihao Liang, Rihui Yao, Honglong Ning, Xiaoqin Wei

**Affiliations:** 1 School of Electrical Engineering, University of South China, Hengyang 421001, China; 2021000103@usc.edu.cn (J.L.); 2020000113@usc.edu.cn (Y.W.); 2 Guangdong Basic Research Center of Excellence for Energy & Information Polymer Materials, State Key Laboratory of Luminescent Materials and Devices, School of Materials Sciences and Engineering, South China University of Technology, Guangzhou 510640, China; 3 Southwest Institute of Technology and Engineering, Chongqing 400039, China; 4 Key Lab of Guangdong Province for High Property and Functional Polymer Materials, South China University of Technology, Guangzhou 510640, China; 5 The International School of Microelectronics, Dongguan University of Technology, Dongguan 523808, China

**Keywords:** high-k, high entropy metal oxide, solution method, TFTs

## Abstract

High-k metal oxides are gradually replacing the traditional SiO_2_ dielectric layer in the new generation of electronic devices. In this paper, we report the production of five-element high entropy metal oxides (HEMOs) dielectric films by solution method and analyzed the role of each metal oxide in the system by characterizing the film properties. On this basis, we found optimal combination of (AlGaTiYZr)O_x_ with the best dielectric properties, exhibiting a low leakage current of 1.2 × 10^−8^ A/cm^2^ @1 MV/cm and a high dielectric constant, while the film’s visible transmittance is more than 90%. Based on the results of factor analysis, we increased the dielectric constant up to 52.74 by increasing the proportion of TiO_2_ in the HEMOs and maintained a large optical bandgap (>5 eV). We prepared thin film transistors (TFTs) based on an (AlGaTiYZr)O_x_ dielectric layer and an InGaZnO_x_ (IGZO) active layer, and the devices exhibit a mobility of 18.2 cm^2^/Vs, a threshold voltage (V_th_) of −0.203 V, and an subthreshold swing (SS) of 0.288 V/dec, along with a minimal hysteresis, which suggests a good prospect of applying HEMOs to TFTs. It can be seen that the HEMOs dielectric films prepared based on the solution method can combine the advantages of various high-k dielectrics to obtain better film properties. Moreover, HEMOs dielectric films have the advantages of simple processing, low-temperature preparation, and low cost, which are expected to be widely used as dielectric layers in new flexible, transparent, and high-performance electronic devices in the future.

## 1. Introduction

Accompanied by the rapid development of science and technology, the development of electronic devices with high integration ability, high response speed, and low power consumption has become a general trend. In this process, the dielectric layer, one of the important components of various electronic devices, has been put forward with low leakage current (<10^−8^ A), high dielectric constant (k > 10), and high transmittance (>85%) requirements [1]. With the miniaturization and high integration ability of electronic devices, the traditional SiO_2_ dielectric layer (with its low dielectric constant) requires reduced thickness, which leads to increased tunneling effect and leakage current. Therefore, it is gradually being replaced by high-k materials (k > 10) [2]. The main factors that researchers focus on when selecting high-K materials include dielectric constant, band gap width, crystallinity, surface morphology, and defect states [3,4]. Currently, a variety of promising high-k dielectric materials have received attention, such as Al_2_O_3_ [5], Ga_2_O_3_ [6], HfO_2_ [7,8], TiO_2_ [9], Y_2_O_3_ [10,11,12], ZrO_2_ [13,14,15], and so on. Among them, HfO_2_, TiO_2_, and ZrO_2_ have the advantage of high dielectric constant (k > 20), but there are problems such as high leakage current and small forbidden bandgap (<5 eV) [16]. Al_2_O_3_, as a common metal oxide dielectric material, is favored because of the advantages of stable performance, good insulating properties, and high crystallization temperature (>800 °C) [17], but the disadvantage of low dielectric constant (k < 10) makes it unable to meet the requirements of high integration of electronic devices. It can be seen that a single metal oxide faces challenges in balancing multiple desirable properties for effective practical application.

By mixing or doping, various high-k materials can be combined to make up for the shortcomings of a single material system, taking into account the performance advantages of a variety of materials, to meet the requirements of electronic devices for high-performance dielectric layers with low leakage current, high dielectric constant, and high transmittance [16,18,19,20,21,22,23]. There have been many studies on the combination of binary or ternary metal oxides, which can be targeted to optimize one of the properties such as dielectric constant, leakage current, or forbidden bandgap. High-entropy metal oxides (HEMOs) contain five or more elements, promising a flexible combination of high-performance metal oxide dielectric materials [24]. High entropy materials are loosely defined as solid solution materials containing more than five dominant elements with equal or nearly equal atomic percentages. The concept of high entropy opens up a new path for the development of advanced materials with unique properties that cannot be achieved by traditional materials approaches based on only one dominant element [25]. Meanwhile, each atom in HEMOs is surrounded by neighboring atoms of different sizes with a highly asymmetric disordered structure, which increases the probability of carrier-atom collisions, effectively reduces the carrier leakage pathway, leading to better dielectric properties [26,27]. Therefore, in this paper, six high-performance dielectric materials with their respective advantages, Al_2_O_3_ for the low leakage current, Ga_2_O_3_ for the wide forbidden bandgap, HfO_2_, TiO_2_, ZrO_2_ for the high dielectric constant and Y_2_O_3_ as carrier inhibitor, were selected to be combined, and six sets of five-element high-entropy combinations were designed for the study, namely, (GaHfTiYZr)O_x_, (AlHfTiYZr)O_x_, (AlGaTiYZr)O_x_, (AlGaHfYZr)O_x_, (AlGaHfTiZr)O_x_, (AlGaHfTiY)O_x_. The solution method is selected for the preparation of five-element high-entropy thin films, which not only has the advantages of a simple process and low cost but also is very convenient for realizing the mixing of multiple elements as well as the proportion regulation [28].

## 2. Materials and Methods

### 2.1. Precursor Solution Preparation and Characterization

2-Methoxyethanol (2-MOE) was chosen as the precursor solvent, and the precursor solutes were selected as aluminum nitrate hydrate (Al(NO_3_)_3_·9H_2_O), gallium nitrate hydrate (Ga(NO_3_)_3_·xH_2_O), hafnium chloride (HfCl_4_), tetrabutyl titanate (C_16_H_36_O_4_Ti), yttrium nitrate hexahydrate (Y(NO_3_)_3_·6H_2_O), and zirconium nitrate (Zr(NO_3_)_4_·5H_2_O), five of which were selected for combination at a time, to configure a high-entropy precursor solution with a concentration of 1.0 M. The molar ratios of the five metal salts were 1:1:1:1:1. We renamed the six-element combinations for clarity, the nomenclature is shown in Table 1. Further optimization we performed by varying the concentration of Ti ions in the AGTYZ fraction. The solvent and solute of the solution were the same as mentioned earlier, but the concentration of the C_16_H_36_O_4_Ti concentrations were adjusted. The total solute concentration in the solution was 1.0 M and the concentration of Ti was increased from 0 M to 0.28 M while the other four solutes were decreased from 0.25 M to 0.18 M. 1% acetic acid was added to the solution to inhibit the hydrolysis of the metal ions. Acetic acid was chosen to avoid the effect of the added acid on the film composition because it’s easy to be removed by heating. After stirring the precursor solution for 12 h, we filtered it with a 13 mm/0.45 μm filter and left it to age for 24 h. The surface tension of the solutions was measured using an optical surface tension meter (Attension T200, Biolin Scientific, Gothenburg, Sweden) and the viscosity was studied using a rheometer (HAAKE MARS 40, Thermo Fisher Scientific, Waltham, MA USA). TG-DSC tests were conducted using a thermogravimetric analyzer (DZ-TGA101, Nanjing Dazhan, Nanjing, China) and a differential scanning calorimeter (DZ-DSC300C, Nanjing Dazhan, Nanjing, China).

### 2.2. Film Fabrication and Characterization

The films were deposited on quartz glass substrates by spin-coating (1000 r/min for 6 s then 5000 r/min for 30 s) with 50 μL of precursor. Next, the films were pre-annealed in the air at 150 °C for 10 min and then annealed at 200 °C, 300 °C, and 400 °C for 1.5 h, respectively. The surface morphology of the films was observed by a laser scanning confocal microscopy (OLS5000-CB, Olympus, Tokyo, Japan) and an atomic force microscope (BY3000, Nano Instruments, Beijing, China). The phase of the films was analyzed using an X-ray diffractometer (EMpyrean X, PANalytical, Almelo, The Netherlands). The optical properties of the films were investigated by a UV-Vis spectrophotometer (UV-2600 Shimadzu, Fukuoka ken, Japan). The thickness of the films was measured by a stylus profiler (Dektak 150, Bruker, Billerica, MA, USA).

### 2.3. Metal-Insulator-Metal Device Fabrication and Characterization

**Figure 1:**
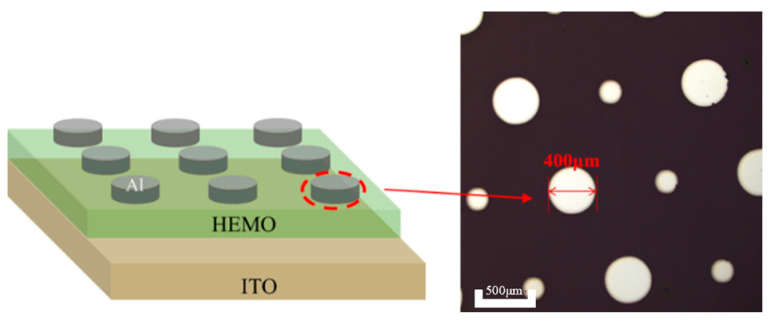
Schematic diagram of MIM device structure.

The electrical properties of HEMOs films were characterized using metal-insulator-metal (MIM) devices with an ITO/HEMOs/Al structure, where HEMOs films were deposited on ITO substrates by spin-coating, and 100-nm-thick Al top electrodes were deposited using thermal evaporation. The device structure is visualized in Figure 1. The current-voltage (I-V), capacitance-frequency (C-F), and capacitance-voltage (C-V) characteristics of the MIM devices were characterized by a semiconductor parameter analyzer (FS-Pro, Primarius Technologies, Shanghai, China).

### 2.4. Thin-Film Transistor Applications and Characterization

HEMOs films were first spin-coated onto T-shaped Al substrates. Subsequently, 20-nm-thick IGZO active layers were deposited via RF pulse sputtering, followed by thermal annealing at 250 °C for 1 h, then 100-nm-thick Al source-drain electrodes deposited through thermal evaporation. Output curves and transfer curves of the TFTs were measured with a semiconductor parameter analyzer (FS Pro, Primarius Technologies, Shanghai, China). 

### 2.5. The Critical Parameter of Thin-Film Transistor

Vth corresponds to the value of the gate voltage when a conductive channel is formed at the active/insulating layer interface. Vth can be obtained by intersecting the Vg axis with the epitaxial part of the linear portion of the I_d_^1/2^. Vth can be obtained by intersecting the Vg axis with the epitaxial part of the linear part of the Id-to-Vg curve in the transfer characteristic.

Mobility (μ) is related to the efficiency of carrier transport in semiconductors and directly affects the maximum current and operating frequency of the device. Mobility can be calculated in different ways. This article calculates the saturation mobility and the formula is shown in (1):

(1) $$\mu =\frac{2L(\frac{d\sqrt{I_{d}}}{\mathrm{dVg}}{)}^{2}}{C_{i}W}$$

The switching ratio (I_on_/I_off_) is defined as the ratio of the open-state current (I_on_) to the closed-state current (I_off_), and the ratio of the maximum current to the minimum current in the transfer characteristic curve is taken in the actual calculation. The inverse value at the maximum slope on the transfer characteristic curve is called SS, as shown in equation (2).

(2) $$\mathrm{SS}=\frac{\partial \mathrm{Vg}}{\partial \log\left(I_{d}\right)}$$

## 3. Results and Discussion

### 3.1. Solution Properties

#### 3.1.1. Rheological Properties

**Figure 2:**
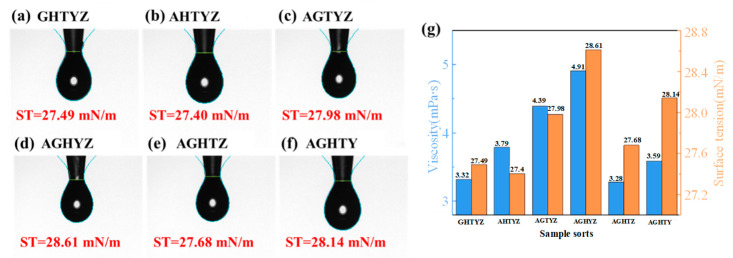
(**a**–**f**) Results of solution ST tests; (**g**) Surface tension and viscosity of precursor solutions.

The rheological properties of the solutions significantly impact the quality of spin-coated film [29]. The results of surface tension (ST) characterization are shown in Figure 2a–g. The ST were slightly decreased compared with the theoretical value of ethylene glycol methyl ether (31.3 mN/m), suggesting that the metal salts reduced the solution’s surface tension. However, varying combinations of elements had minimal effects on the surface tension. As shown in Figure 2g, the ST of all six solutions were lower than 30 mM/m, the solution viscosities were lower than 5 mPa∙s, and the rheological properties of the precursor solutions were in the range suitable for the spin-coating method [30].

#### 3.1.2. Chemical Properties

**Figure 3:**
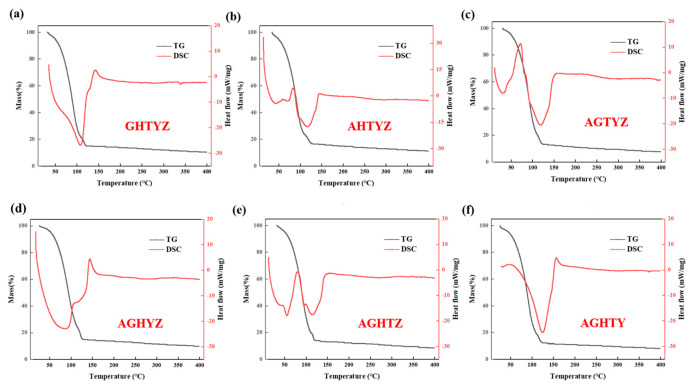
TG-DSC characterization results of solutions: (**a**) GHTYZ solution; (**b**) AHTYZ solution; (**c**) AGTYZ solution; (**d**) AGHYZ solution; (**e**) AGHTZ solution; (**f**) AGHTY solution.

The solutions were subjected to TG-DSC tests, and the results are shown in Figure 3a–f. The TG curves of the solutions showed similar trends. They had an obvious mass loss in the temperature interval from room temperature to 125 °C, which was due to the rapid evaporation of the solvent, ethylene glycol methyl ether. In addition, it could be observed that the TG curves exhibited inflection points between 100 °C and 125 °C, accompanied by a decreased rate of mass loss. This phenomenon can be attributed to the significant increase in solution concentration after substantial solvent evaporation, strengthening the binding force between the remaining solvent molecules and the solute molecules, thus requiring more energy for molecules to escape from the solution system [31,32]. Above 125 °C, the mass loss rate decreased, which may result from the continued decomposition of organic residuals and acid groups [33]. The presence of heat absorption peaks around 50 °C in some curves may be due to desorption of adsorbed moisture [34,35]. The endothermic peaks around 150 °C observed in some curves may be associated with the hydrolysis of metal cations forming hydrogen bonds and the formation of reticulated structures through metal ion coordination. Based on the TG-DSC analysis, the optimal annealing temperature should exceed 150 °C. Changes in the components of the solution during heating were detected by TGDSC tests. Lower temperatures may result in incomplete organic solvent removal, hindering the formation of dense and uniform films, while excessive temperatures could promote crystallization, increasing device leakage current. Therefore, the annealing temperatures were first set to 200 °C, 300 °C, and 400 °C to further optimize the temperature.

### 3.2. Films Properties

#### 3.2.1. Physical Properties

**Figure 4:**
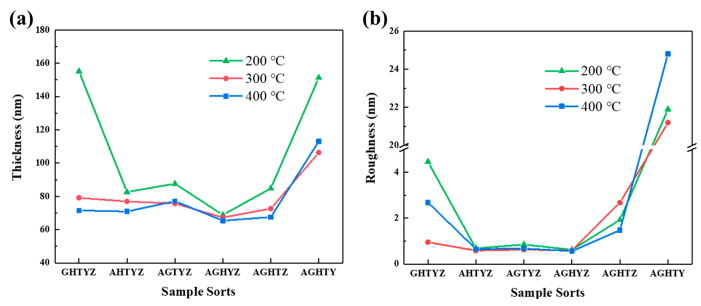
(**a**) Thickness of HEMOs films; (**b**) roughness of HEMOs films.

The film thickness characterized by the stylus profiler and the roughness measured by the AFM are shown in Figure 4a and Figure 4b respectively. The film thickness and surface roughness exhibit similar trends with the change of components. While temperature is higher than 200 °C, except for the AGHTZ and AGHTY, the film thickness ranged from 70 to 80 nm, and the Root Mean Square of the roughness (Sq) of the films is lower than 2 nm. At an annealing temperature of 200 °C, the films generally show increased thickness, especially GHTYZ and AGHTY. As the annealing temperature increases to 300 °C and 400 °C, the film thickness stabilizes, indicating that temperature ceases to be the dominant factor affecting film thickness above 300 °C. At this time, the AGHTY film still shows abnormally high film thickness, combined with the film roughness test results shown in Figure 4b, the AGHTY film also shows abnormally high surface roughness (Sq *>* 20 nm).

**Figure 5:**
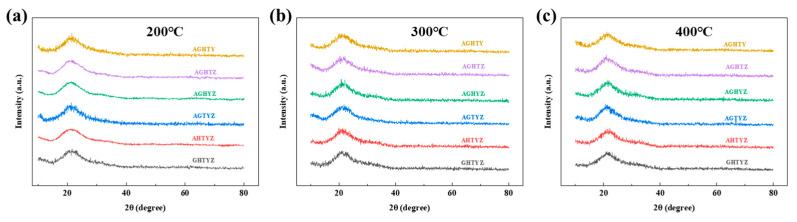
XRD test results of HEMOs films with different annealing temperatures: (**a**) 200 °C; (**b**) 300 °C; (**c**) 400 °C.

The films annealed at different temperatures were subjected to XRD tests as shown in Figure 5a–c. Only the diffraction peaks of the glass substrate can be measured around 2θ ≈ 21°, which indicates that all the high-entropy oxide films are mainly amorphous even when the annealing temperature is up to 400 °C. According to some previous studies, the crystallization temperature of ZrO_2_ and HfO_2_ is low (about 350 °C), while Al_2_O_3_ and Ga_2_O_3_ have high crystallization temperatures. Therefore, the additions of Al_2_O_3_ and Ga_2_O_3_ can effectively increase the overall crystallization temperature of the films. Amorphous film is favorable for large-area preparation and can inhibit the formation of current channels, which helps reduce device leakage current and device power consumption [36]. Based on the comprehensive analysis of both the solutionand film properties, different annealing temperatures were investigated. At 200 °, organic residuals and acid ions can not be completely removed from the films, degrading the film density and uniformity. When the annealing temperature was increased from 300 °C to 400 °C, no significant improvement in film morphology was observed. Considering both the cost-effectiveness of the preparation process and the requirement of low-temperature deposition for flexible electronics, an annealing temperature of 300 °C was selected for further experiments.

Here the physical properties of the film have been analyzed, such as film thickness, roughness and crystallinity. From the test results, except for AGHTYZ and annealing temperatures above 200 °C, the component films obtain a good surface property and are in an amorphous state.

#### 3.2.2. Optical Properties

**Figure 6:**
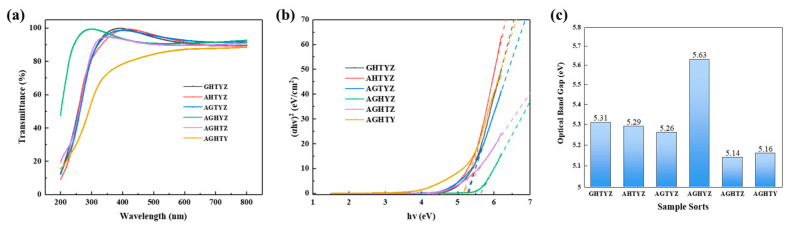
Results of film optical characterization: (**a**) transmittance; (**b**) (ahν)^2^-hν curves; (**c**) optical bandgap of HEMOs films.

The optical properties of the high entropy metal oxide films were characterized by a UV-Vis spectrophotometer. The transmittance of the films are shown in Figure 6a. All the films containing Zr show good visible light transmittance (over 89% in the 380*–*380 nm range), which shows their potential to be used in transparent electronic devices. As shown in Figure 6b, the optical band gaps were derived by linear fitting the (ahν)^2^-hν curves. The optical bandgap of the films is shown in Figure 6c. Since the high-entropy system contains a variety of materials with high forbidden bandgap, the HEMOs films exhibit large optical bandgaps (*>*5 eV), with the AGHYZ films having the largest optical bandgap (~5.63 eV). The bandgap of TiO_2_ (~3.5 eV) is the smallest among six oxides, thus the optical bandgap of the HEMOs films containing TiO_2_ is reduced.

The films were characterised by high transmittance and high bandwidth through transmittance testing and bandwidth fitting, which facilitates the obtaining of a dielectric film with a low leakage current.

#### 3.2.3. Components Analysis

**Figure 7:**
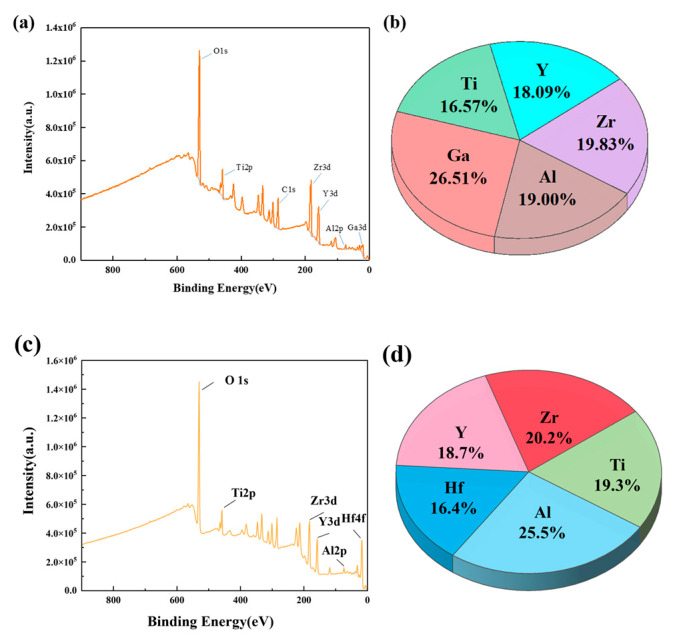
(**a**) XPS full spectrum of the AGTYZ film; (**b**) the percentage of each element in the AGTYZ film; (**c**) XPS full spectrum of the AHTYZ film; (**d**) the percentage of each element in the AHTYZ film.

XPS test was conducted to acquire information on elemental content and chemical states. XPS analysis of the AGTYZ film revealed characteristic peaks for Al2p, Ga3d, Ti2p, Y3d, and Zr3d, along with the percentage of each element in the AGTYZ, as shown in Figure 7a,b. The observed valence states of these 5 metal elements correspond to the typical oxidation states in their common oxides. A similar situation occurs with AHTYZ films, as shown in Figure 7c,d. The distribution of the six metal oxides in the films has been obtained by XPS of these two compositions. It showed that each element constitutes approximately 20% of the film composition, consistent with the intended stoichiometry. This confirms that multi-elemental HEMOs dielectric layers can be successfully synthesized via solution processing, and the proportion of each content can be easily controlled.

**Figure 8:**
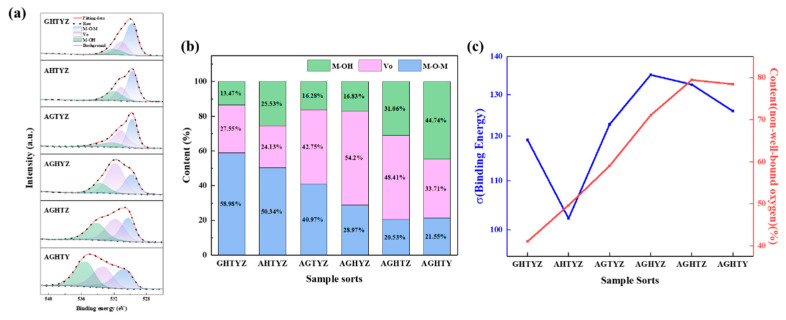
(**a**) XPSO1s spectra of HEMOs films; (**b**) percentage of three types of oxygen chemical states of HEMOs films; (**c**) the average difference in oxygen binding energy and the percentage of non-well-bound oxygen of HEMOs films.

The O1s XPS spectra were deconvoluted into three peaks with a Gaussian distribution, as shown in Figure 8a. The peaks centered at 529.7 eV, 531.1 eV, and 532.2 eV correspond to metal–oxygen bonds (M-O-M), oxygen vacancies (V_o_), and hydroxyl groups (M-OH), respectively, formed through water adsorption and related processes [37]. The proportions of the three oxygen chemical states in HEMO films are summarized in Figure 8b. The highest content of M-O-M bonds of 58.98% was found in the GHTYZ film. The relatively low proportion of M-O-M bonds and the significant proportional variation among different combinations are postulated to be the results of the oxygen chemical states being strongly influenced by the complex internal structure of HEMOs; furthermore, larger metal–oxygen binding energy differences among elements may result in more oxygen vacancies [38]. To investigate this phenomenon, the average binding energy differences for six HEM combinations were calculated using reported values for individual metal–oxygen bonds [39,40,41,42,43,44]. Figure 8c reveals a correlation between the average binding energy differences and the proportion of M-O-M bonds in the films. The larger the average difference in binding energy of the metal–oxide bonds in the HEMOs films, the more complex the internal structure of the film is, increasing the proportion of non-well-bound oxygen. Based on the above analysis, the complexity of the internal structure of HEMO films can be increased by increasing the average difference of the binding energy, which suppresses the formation of conductive channels and lowers the device leakage current. However, excessive structural complexity can also lead to the formation of more oxygen-related defects, resulting in an increased leakage current.

The individual metal oxides were uniformly distributed in the film by XPS testing of the film. Moreover, the oxygen vacancies of the films were analysed. High entropy metal oxides usually have a low M-O-M content due to their complex structure. The complex structure hinders the formation of conductive channels and thus reduces the leakage current. However, the defects brought by the complex structure may also increase the leakage current. Therefore, it needs to be further analysed in conjunction with thin film leakage current testing.

### 3.3. Devices Properties

#### 3.3.1. MIM Properties

**Figure 9:**
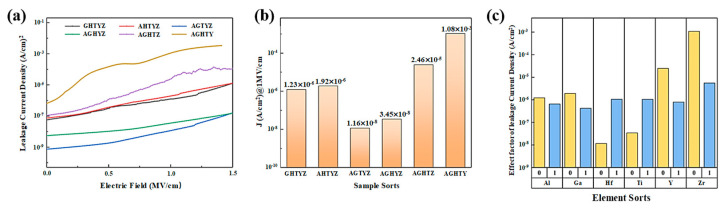
Film I-V characteristics: (**a**) I-V curves; (**b**) leakage current density under an electric field of 1 MV/cm; (**c**) factor analysis of each element’s impact on leakage current density.

The current-voltage (I-V) curves of the MIM devices and the leakage current density @1 MV/cm of the films are shown in Figure 9a,b. The elemental composition in the high entropy film exhibited a significant influence on the leakage current density of the film. AGHTZ and AGHTY films displayed large leakage current densities (*>*1 × 10*^−^*^5^ A/cm^2^@1 MV/cm). These two groups of films have a rough surface as well as loose internal structure, with only approximately 20% of well-bonded oxygen content. The presence of a large number of oxygen-related defects primarily contributed to the increase in leakage current density. The AGTYZ film exhibited the lowest leakage current density (1.2 × 10*^−^*^8^ A/cm^2^@1 MV/cm), which is conjectured to its larger average difference in binding energy in the film and lower percentage of non-well-bound oxygen.

In factor analysis, each element’s impact was quantified by comparing the average property values between compositions with and without the specific element. The factor of each element and property was calculated as follows: To determine the impact of element X on property Y, the average value of property Y for all compositions containing X (designated as X = 1) was compared with the average value of property Y for all compositions without X (designated as X = 0). A factor value of X = 1 indicates that the element enhances the specific property under investigation, while a factor value of X = 0 suggests an inhibitory effect. This systematic analysis enables the isolation and quantification of each element’s contribution to each property of HEMO films.

Factor analysis was performed to evaluate the impact of six metal oxide (AlO_x_, GaO_x_, HfO_x_, TiO_x_, YO_x_, and ZrO_x_) on the leakage current density in HEMOs films, as presented in Figure 9c. The presence of AlO_x_ and GaO_x_ was found to reduce the leakage current density. The introduction of Al helps to reduce the film roughness and the interface trap density, while the wide forbidden bandgap of Al_2_O_3_ suppresses the electron leap. Meanwhile, Ga_2_O_3_ has a large positive Gibbs free energy, which can effectively suppress the deterioration of the film dielectric properties caused by the hygroscopic reaction [6]. Hf and Ti addition significantly increased the leakage current density of HEMOs films, which can be attributed to the residual chloride ions, nitrate ions, and organicsintroduced in the precursor solutions (HfCl_4_, C_16_H_36_O_4_Ti, EGME) require higher thermal energy for decomposition and remain partially undecomposed after annealing, creating additional electron transport pathways in the dielectric films. However, the large positive Gibbs free energy of TiO_2_ may promote film deterioration through hygroscopic reaction, while the narrow forbidden bandgap of TiO_2_ facilitates electron leap in the films, increasing leakage current density in HEMOs films [45].

**Figure 10:**
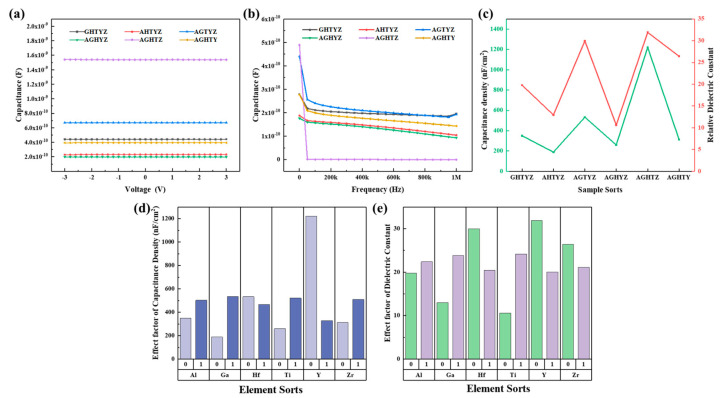
(**a**) C-V curves; (**b**) C-F curves; (**c**) Capacitance density, relative dielectric constant; (**d**) factor analysis of each element’s impact on capacitance density; (**e**) factor analysis of each element’s impact on dielectric constant.

The capacitance-voltage (C-V) and capacitance-frequency (C-F) curves are shown in Figure 10a,b. Under a 1000 Hz electric field, the capacitance density and dielectric constant of HEMOs films were investigated, as summarized in Figure 10c. From the C-V curves, it can be observed that under a fixed-frequency electric field, the capacitance of the film is almostconstant with varying applied voltage, showing good voltage stability. The C-F measurements up to 1 MHz demonstrate that the capacitance decreases as frequency increases, suggesting a decrease in capacitance density and dielectric constant. This can be attributed to the polarization in the dielectric layer under an alternating electric field, where the dielectric constant is closely related to different types of polarization occurring at various frequencies within the film [46]. At low frequencies (<10^2^ Hz), almost all polarization mechanisms can respond to the change in electric field, resulting in higher polarization and consequently larger dielectric constant. As the frequency increases, some polarization processes fail to keep pace with the field variations, decreasing polarization and then the dielectric constant [47].

The HEMOs films exhibit a highly disordered and asymmetric internal structure due to the incorporation of various smetallic elements with different atomic radii, enhancing atomic polarization under external electric field, contributing to higher dielectric constants. Among the studied films, AGHTZ film exhibits the largest capacitance density and dielectric constant at low frequencies, but undergoes rapid degradation with increasing frequency. The capacitance decreases directly from 4.89 × 10*^−^*^10^ to 1.28 × 10*^−^*^12^ at frequencies above 1000 Hz. As depicted in Figure 9b, the AGHTZ film exhibits a large leakage current density, possibly due to residual nitrates, hydroxides, or organic groups, as well as potential absorption of moisture from the air, increasing mobile charges. The presence of this mobile charges increase the overall polarization in the low-frequency range, but the capacitance-frequency stability decreases in the mid to high-frequency range. To better analyze the role of different elements in the HEMOs films on the dielectric properties, the effects of six metal oxide, AlO_x_, GaO_x_, HfO_x_, TiO_x_, YO_x_, and ZrO_x_, on the capacitance density and dielectric constant of the films were under factor analysis, as shown in Figure 10d,e. The result indicates that Ti significantly enhances capacitance density and dielectric constant, consistent with the high dielectric constant (60~80) in previous studies [16].

Considering all the above analysis, it leads to a conclusion that good surface properties result in excellent dielectric properties. YO_x_ and ZrO_x_ show good compatibility with other metal oxides, forming HEMOs film with good surface morphology. This superior quality also facilitates the formation of a dielectric HEMO layer and metal oxide semiconductors with low interface trap density. The wide bandgap of Al_2_O_3_ suppress the electron leaps within HEMOs film, while the large positive Gibbs free energy of Ga inhibits the degradation of dielectric properties caused by hygroscopic reaction. Additionally, the high crystallization temperature of Al_2_O_3_ and Ga_2_O_3_ enables the HEMOs film to maintain an amorphous state after 400 °C annealing, thereby reduces the formation of conductive channelsand the leakage current. Among 6 components, TiO_2_ has the largest dielectric constant, significantly enhancing the dielectric constant of HEMOs films. The optimized films demonstrated excellent optical and electrical properties, including a high visible light transmission of 93.8%, low leakage current density of 1.2 × 10*^−^*^8^ A/cm^2^@1 MV/cm, a high dielectric constant of 29.95 at 1000 Hz, and good frequency stability.

Previous studies have shown that while single TiO_2_ dielectric layers exhibit high dielectric constants (k: 60*–*80), their relatively low bandgap (3.45 eV) leads to large leakage currents, limiting their application as high-performance dielectric layer. However, the AGTYZ films containing Ti maintain both a high dielectric constant and a high optical bandgap of 5.26 eV. To further investigate the role of Ti in modulating the dielectric constant and optical bandgap, the Ti concentrations in AGTYZ films were varied, as detailed in Table 2.

**Figure 11:**
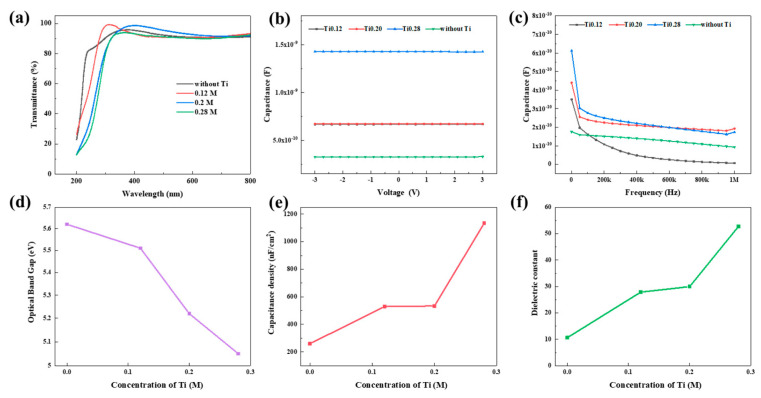
Effect of Ti concentration on film properties: (**a**) transmittance; (**b**) C-V curves; (**c**) C-F curves; (**d**–**f**) Plot of Ti concentration versus optical band gap, capacitance density, dielectric constant.

The optical and capacitive properties of HEMOs films with different concentrations of Ti were measured, as shown in Figure 11a–c. The optical bandgap of the films was fitted, the capacitance density and dielectric constant were calculated, as shown in Figure 11d–f. Results indicate that Ti concentration significantly modulates both optical band gap and dielectric properties of the films. Specifically, Ti concentration exhibits an inverse relationship with the optical band gap and a positive correlation with capacitance density and dielectric constant, confirming our previous factor analysis results. As the concentration of Ti increases from 0 to 0.28 M, the capacitance density of the films increases from 259.72 nF/cm^2^ to 1133.50 nF/cm^2^, and the dielectric constant rises significantly from 10.63 to 52.74, while the optical band gap shows a modest decrease from 5.62 eV to 5.05 eV. These findings suggest that adjusting Ti content in AGTYZ film enables dielectric HEMO layers to combine high dielectric constant with wide optical bandgap.

By comparing the performance of MIM devices prepared from six metal oxides, including leakage current and dielectric constant, we found that AGTYZ has the best performance. And in the factor analysis, Ti has the effect of increasing the dielectric constant, so we investigate the effect of Ti content in AGTYZ on the film properties. The dielectric constant of the final dielectric film increased to 52.74.

#### 3.3.2. TFT Properties

**Figure 12:**
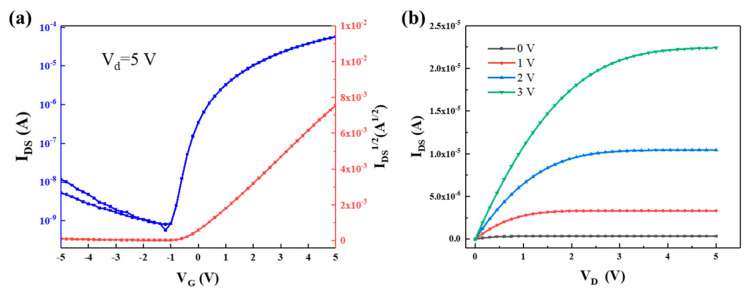
TFT performance characterization: (**a**) TFT transfer curve and and I_DS_^1/2^-V_G_ curve; (**b**) TFT output curve.

Given their excellent surface morphology and dielectric properties, TFT devices with a magnetron-sputtered IGZO active layer and an AGTYZ dielectric layer were prepared. The device characteristics are shown in Figure 12a,b. The TFTs exhibited a switching ratio of 1.0 × 10^5^, a mobility of 18.2 cm^2^/Vs, am SS of 0.288 V/decade, and a V_th_ of *−*0.203 V. More importantly, they exhibited a minimal hysteresis effect. In general, the cause of the hysteresis effect is theorized to be carrier trapping at defect sites within the active layer or at interfaces between layers [48]. These results demonstrate that AGTYZ films possess low defect density and high-quality interfaces between the active and dielectric layers. As shown in Table 3, in contrast to other TFTs prepared with single-component dielectric layers, the TFT with a dielectric AGTYZ layer investigated in this study exhibits higher mobility, a higher switching ratio, a lower operating voltage, and subthreshold swing. By combining the advantageous properties of Al_2_O_3_, Ga_2_O_3_, TiO_2_, Y_2_O_3_, and ZrO_2_, high-performance dielectric HEMO layers were successfully developed and implemented in thin-film transistors.

## 4. Conclusions

In this paper, five-element HEMO films were prepared by spin coating, and the effects of the components on the properties of HEMO films were investigated. The results show that (AlGaTiYZr)O_x_ film exhibits the best dielectric properties among the six investigated five-element HEMOs, with more than 90% visible transmittance, an optical bandgap of 5.26 eV, a leakage current density of 1.2 × 10^−8^ A/cm^2^ at a field strength of 1 MV/cm, and a high dielectric constant of 29.95 under a 1000 Hz electric field. On this basis, we prepared IGZO TFTs with dielectric HEMO layers to investigate the suitability of HEMO films for TFTs. The test results show that the TFTs based on a dielectric (AlGaTiYZr)O_x_ layer exhibit a mobility of 18.2 cm^2^/Vs, a V_th_ of −0.203 V, and an SS of 0.288 V/decade, along with a minimal hysteresis, suggesting the potential of applying HEMOs to TFTs. In addition, we factorized the performance parameters of the HEMO films and found that Ti has a significant enhancing effect on the dielectric constant. Based on this, we achieved the modulation of the dielectric constant by adjusting the ratio of Ti in the HEMO films, increasing the dielectric constant to 52.74 and maintaining a large optical bandgap of 5.05 eV. Overall, dielectric HEMO films prepared by spin coating can synthesize the advantages of multiple high-k dielectrics to obtain better film properties while having the advantages of simple processing, low-temperature preparation, and low cost. HEMO films are expected to be widely used as dielectric layers in the future for flexible, transparent, and high-performance electronic devices.

## Figures and Tables

**Table 1:** HEMOs sample nomenclature.

Sample Names	(GaHfTiYZr)O_x_	(AlHfTiYZr)O_x_	(AlGaTiYZr)O_x_	(AlGaHfYZr)O_x_	(AlGaHfTiZr)O_x_	(AlGaHfTiY)O_x_
Abbreviation	GHTYZ	AHTYZ	AGTYZ	AGHYZ	AGHTZ	AGHTY

**Table 2:** Ti concentration settings.

Elemental Components	The Concentration of Ti (M)	The Concentration of Remaining Elements (M)	Total Concentration (M)
AGTYZ	0	0.25	1.0
	0.12	0.22	
	0.20	0.20	
	0.28	0.18	

**Table 3:** Comparison between AGTYZ TFTs and other single component TFTs.

Dielectric	Ref.	Mobility (cm^2^/Vs)	I_on/_I_off_	Vth (V)	SS (V/dec)
AGTYZ		18.2	10^5^	−0.203	0.288
Al	[49]	4.7	10^3^	0.2	0.24
Ga	[50]	3.09	10^5^	0.83	0.18
Ti	[51]	0.2	10^3^	−24	/
Y	[52]	34	10^5^	−4	/
Zr	[53]	12.7	10^5^	0.6	0.36

## Data Availability

The raw data supporting the conclusions of this article will be made available by the authors on request.
